# A Case Report of a Baby Born to a Woman With Isolated Right Ventricular Apical Hypoplasia Without an Atrial Septal Defect

**DOI:** 10.7759/cureus.51764

**Published:** 2024-01-06

**Authors:** Hiroaki Kawano, Yutaro Seo, Hiroki Usuku, Fumi Oike, Megumi Nakata, Shota Nakamura, Michiko Sugita, Kenichi Tsujita

**Affiliations:** 1 Department of Cardiovascular Medicine, Graduate School of Medical Sciences, Kumamoto University, Kumamoto, JPN; 2 Department of Obstetrics and Gynecology, Graduate School of Medical Sciences, Kumamoto University, Kumamoto, JPN; 3 Department of Anesthesiology, Graduate School of Medical Sciences, Kumamoto University, Kumamoto, JPN

**Keywords:** adult congenital heart disease, right heart failure, pregnancy and heart disease, giant right atrium, hypoplastic right ventricle

## Abstract

Pregnancy is a major life event for most women that causes extensive physiological changes. Hence, it is associated with additional risks in women with congenital heart disease. No reports of pregnancy or a baby born to a woman with isolated right ventricular hypoplasia without an atrial septal defect have been published. In this case, the patient’s right ventricle was very small with no contractility. The right atrium was highly enlarged, and its contractility resulted in pulmonary circulation without pulmonary hypertension. The size increased post-delivery than that before pregnancy. Fortunately, a healthy infant was born without any right heart failure symptoms.

## Introduction

A lot of patients born with congenital heart disease (CHD) survive to adulthood due to improvements in cardiology care [1]. Pregnancy is a major life event for most women that causes physiological changes in the body. Thus, it is associated with additional risks in women with CHD. Subsequently, heart failure, arrhythmias, and worsening cardiac conditions may expose the mother and infant to an increased risk of morbidity and mortality. We think cardiologists, obstetricians, anesthesiologists, and pediatricians must be aware of the types of CHD encountered in adults and the potential risks to the mother, fetus, and neonate during pregnancy. A multidisciplinary approach to managing pregnancy in these women is essential.

We have reported a patient with isolated right ventricular hypoplasia without an atrial septal defect [2]. To the best of our knowledge, there are no other reports of patients with isolated right ventricular hypoplasia without an atrial septal defect growing to adulthood. No reports of pregnancy or babies born in such rare cases have been published. Here, we show the pregnancy and a baby born in this rare case.

## Case presentation

**Figure 1:**
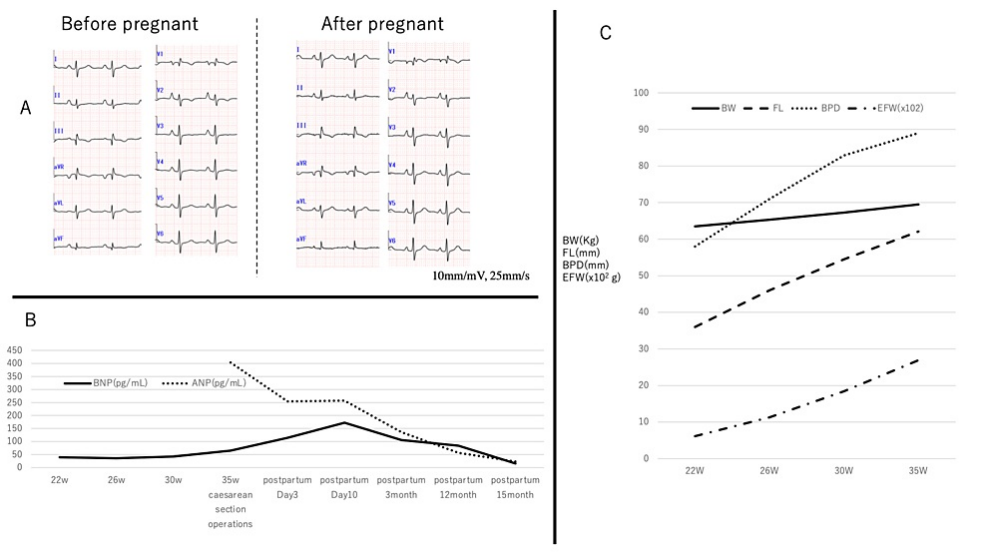
Mother's electrocardiogram (ECG), A-type, B-type natriuretic peptide, and fetal data changes Panel A: There are no changes in ECG before and after pregnancy. Panel B: Changes of A-type and B-type natriuretic peptide during pregnancy. Panel C: Changes of mother‘s body weight (BW), fetal femur length (FL), biparietal diameter (BPD), and estimated weight (EFW) during pregnancy.

**Figure 2:**
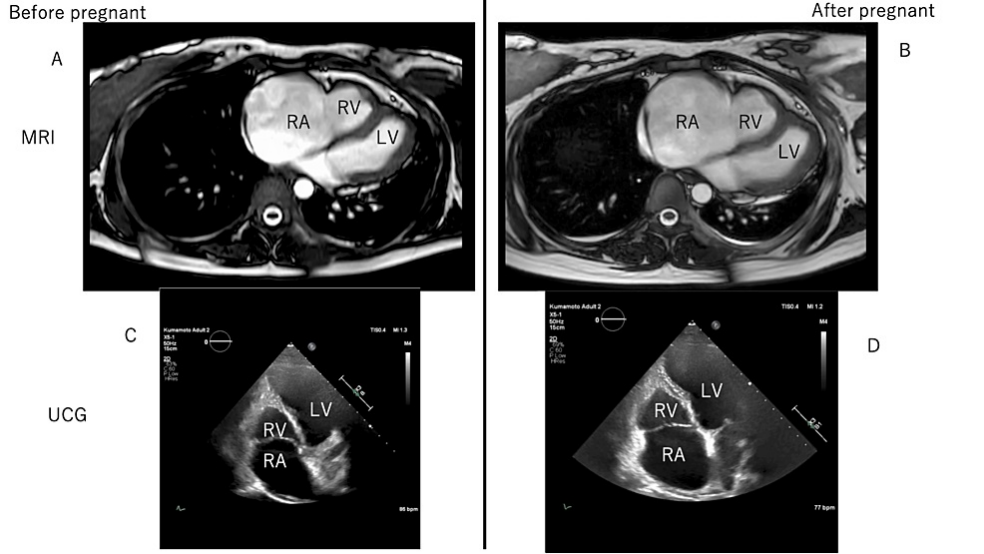
Right heart changes before and after pregnancy in magnetic resonance images (MRI) and transthoracic echocardiography (TTE) LV: left ventricle RA: right atrium RV: right ventricle Panel A: before pregnancy MRI image   Panel B: after pregnancy MRI image Panel C: before pregnancy TTE image     Panel D: after pregnancy TTE image

The patient’s age was 28 years old (height 160 cm, body weight 60 Kg, BP 122/79). In this case, pregnancy may aggravate right-sided heart failure in mothers, leading to fetal growth impairment. The patient and her husband aspired to conceive a child and pursued infertility treatment, resulting in her eventual pregnancy. It is possible that the circulating blood volumes of the mother's body increased as the pregnancy progressed, leading to right heart failure. Her right ventricle was very small, with no contractility [2]. Thus, the right atrium was very large. Increased right atrial blood pressure and contractility resulted in increased right heart circulation. Augmented positive P waves in I II V2-6 and negative P waves in aVR in electrocardiography (ECG) may indicate a large right atrium (Figure 1A). Thus, there was a high risk for atrial fibrillation (AF) onset. Throughout the pregnancy, we conducted a comprehensive maternal health assessment, which encompassed ECG, transthoracic echocardiography (TTE) with measurements of vena cava diameter during expiration and inspiration, and plasma B-type natriuretic peptide (BNP) levels. Plasma A-type natriuretic peptide (ANP) levels were measured after cesarean delivery. ANP and BNP levels were measured using a chemiluminescent microparticle immunoassay (Abbott Architect ci8200, Abbott Laboratories, Abbott Park, IL, USA). Fortunately, we did not observe any edema, high blood pressure, or proteinuria during pregnancy. Neither the ECG findings nor the diameter of the vena cava changed (Figure 1A). TTE showed hepatic vein and inferior vena cava dilation. BNP levels increased as the pregnancy progressed (Figure 1B). The fetus grew well (Figure 1C). At 35 weeks of pregnancy, the fetus may grow sufficiently to live outside the mother’s body. The mother did not exhibit any symptoms of right-sided heart failure symptoms. These findings led us to decide to perform a cesarean delivery under general anesthesia. We used elastic stockings on both lower limbs to limit sudden increases in venous return to the right heart. Central venous pressure (CVP) was approximately 30-35 mm Hg before the cesarean section operation. After the delivery, the CVP decreased to 25-30mm Hg. Fortunately, both the mother and baby (body weight, 3200 g) were healthy. Magnetic resonance imaging and TTE revealed that the right atrium enlarged after the baby was born (Figure 2). TTE showed that the RV area was diastole 10.9/systole 8.8cm^2^ in before pregnancy (Figure 2C) and was diastole 11.5/ systole 8.3 cm^2^ in after cesarean delivery (Figure 2D). BNP and ANP levels peaked around the time of the cesarean delivery. The levels were improved at one year and three months after the operation. Both the maternal and neonatal individuals displayed a preference for breastfeeding provided by the mother. The infant received attentive care from both the mother and her husband.

## Discussion

Usually, counseling and risk prediction are offered to women with CHD at the start of pregnancy. We also counseled her, her husband, and her family about the risk of pregnancy several times. Nonetheless, the patient and her husband remained steadfast in their desire to conceive. Subsequently, she sought infertility treatment at a different medical facility without disclosing any information about her congenital heart condition and successfully became pregnant. Consequently, our medical team provided comprehensive support to the patient and her family in their journey to parenthood. Right ventricular (RV) dysfunction leads to changes in venous pressure, resulting in venous congestion [3]. This case showed isolated right ventricular apical hypoplasia without an atrial septal defect. Her right ventricle was very small, with no contractility [2]. The right atrium was enlarged, which increased blood pressure, and contractility of the right atrium resulted in pulmonary circulation without pulmonary hypertension. RV dysfunction appears to affect placentation, resulting in an increased resistance to uterine artery flow. This condition is associated with pregnancy complications [4]. Diminished RV dysfunction increases the risk of deterioration during pregnancy. Women with systemic right ventricle function should be advised against pregnancy, as in the present case [5]. CVP was very high during cesarean section (gestational age: 35 weeks). In this particular case, CVP may have represented the threshold of gestational duration beyond which significant right-sided heart failure did not occur. Fortunately, the fetal growth was favorable. No exacerbation of right heart failure symptoms was observed in the mother. Infant delivery is a major problem, especially in cases of CHD in adults. Because of the high risk of pregnancy, we chose to perform cesarean section operations under general anesthesia with CVP monitoring. If AF occurs, the mother’s hemodynamics may suddenly develop owing to the absence of an atrial septal defect, resulting in a critical condition. In this case, plasma BNP levels varied below 100 pg/mL during the gestational period. Singh and co-workers reported that women with cardiac disease and plasma BNP levels of 98 pg/ml or lower do not experience any adverse events [6]. Both plasma ANP (302.6pg/mL) and BNP peaked (256.4pg/mL) at eight days postpartum and decreased slowly over time, without any heart failure symptoms. Unfortunately, we started to measure ANP after the cesarean delivery. The right atrium became larger after the baby was born than before pregnancy. These data may indicate that right atrial pressure increases during pregnancy. The long time required to normalize both plasma ANP and BNP levels may indicate that the pregnancy caused cardiac stress or damage. A healthy baby was born without any major problems in the mother’s body. However, this patient may have been fortunate in her outcome.

## Conclusions

A baby was born to a woman with isolated right ventricular apical hypoplasia without an atrial septal defect. Fortunately, a healthy infant was born without any right heart failure symptoms. This patient may have been fortunate in her outcome.
